# Effect of Suture Type and Suture Distance on Holding Strength in Nasal Septal Laceration Model

**DOI:** 10.4274/tao.2021.6100

**Published:** 2021-03-26

**Authors:** Alper Köycü, Evren Hızal, Ozan Erol, Adnan Fuat Büyüklü

**Affiliations:** 1 Department of Otolaryngology, Head and Neck Surgery, Başkent University, Ankara, Turkey

**Keywords:** Holding strength, nasal septum, septal perforation, suture distance, suture material, cadaveric animal study

## Abstract

**Objective:**

Septal mucosal-perichondrial flaps can be lacerated during the elevation of the flaps. Appropriate repair of the lacerations is essential to prevent the development of septal perforation during the healing process. We aimed to determine the superior suture type and suture distance to use in repairing the lacerations of nasal septal mucosal-perichondrial flaps.

**Methods:**

The study used 128 nasal septal mucosal-perichondrial flaps prepared from sheep heads. Experimentally induced lacerations on the mucosal-perichondrial flaps were sutured with two interrupted sutures using one of four suture materials (4-0/5-0 Polyglactin 910, 4-0/5-0 Polydioxanone) and leaving either 5 mm or 10 mm distance between the sutures. Maximum tissue holding strength (HS_max_) was measured for each suture material and suture distance used.

**Results:**

Mean HS_max_ values were higher for Polyglactin 910 sutures (p<0.001) and 10 mm suture distance (p=0.008) when the groups were compared in terms of suture material and suture distance, respectively. There was no statistically significant difference between the mean HS_max_ values of sutures with 4-0 and 5-0 diameters (p=0.057).

**Conclusion:**

Polyglactin 910 suture material with 10 mm space between two adjacent sutures may be more durable than the other suture materials when repairing nasal septal mucosal lacerations.

## Introduction

Nasal septal perforation is one of the complications of septorhinoplasty that can lead to uncomfortable symptoms such as crusting, a feeling of nasal obstruction and epistaxis (1). Closure of the nasal septal perforation generally requires additional surgery that is technically difficult, and even if surgical closure is successful, nasal symptoms may persist even in the long-term follow-up (2, 3). Therefore, all necessary measures should be taken to prevent the development of septal perforation.

During septorhinoplasty, unwanted tears of the septal mucosal perichondrium and/or periosteum may occur. Especially in revision septorhinoplasty cases, septal mucosal-perichondrial flaps can be lacerated during the elevation of the flaps. Appropriate repair of the lacerations is essential to prevent the development of septal perforation during the healing process. For a successful repair, the wound edges should be approximated end-to-end with minimal tissue tension. However, it can be hard to achieve tension-free closure, especially in revision cases. As the intranasal area is narrow and the surgeon has limited space for maneuvers, the repair of nasal septal mucosal-perichondrial lacerations can be quite challenging, especially when the gap between the wound edges is large. In addition, the sutures must be able to remain in the correct position until mucosal healing is completed, without opening, breaking, or causing secondary mucosal tears. In attempt to overcome technical difficulties in septal mucosal-perichondrial suturation, various suture materials and techniques have been developed (4, 5, 6). However, the effect of different suture materials and/or techniques on wound closure and prevention of septal perforation remains controversial.

Tensile strength is the maximum power of the suture resisting breakage. Holding strength, on the other hand, is defined as the amount of force that results in violation of the tissue or slippage of the suture (7). Together with the tensile strength of the suture, the holding strength of the tissue is also important to achieve stable wound closure. Approximation of the wound edges in nasal septal lacerations may result in tension in the flaps, making the holding strength of the flaps critical for the successful repair of the defect. There are studies in the literature that have measured the holding strength in various tissues such as the kidney (7, 8). To the best of our knowledge, the holding strength of nasal septal mucosa is unknown. In addition, the effects of the suture material and the distance between the sutures on the holding strength in nasal septum mucosa are yet to be established.

The aim of this study was to measure the holding strength of nasal septal mucosal-perichondrial flaps that have been experimentally lacerated and repaired with various suture materials applied at different spatial intervals.

## Methods

This experimental study was conducted between March and May 2018, at Başkent University Hospital, Department of Otolaryngology, Head and Neck Surgery. This study was approved by Ba***ş***kent University’s Institutional Review Board (project no: DA18/09, date: 18.09.2018). Seventy heads of slaughtered young adult merino sheep (6–12 months of age) were obtained from a butcher. Sheep nasal septums that were unintentionally torn during submucosal-perichondrial elevation were excluded from the study. A total of 128 nasal septal mucosal-perichondrial flaps prepared from the 64 heads of sheep were used. The flaps were lacerated and repaired with various suture materials applied at different spatial intervals. The maximum holding strength (HS_max_) of the sutured flaps were then measured and compared with each other.

The sheep heads were stored at a temperature of 2–4 °C for conservation until the procedures were performed and frozen specimens were not used. All test procedures were performed at room temperature within 24 hours of each animal’s death.

### Preparation of Nasal Septal Mucosal-Perichondrial Flaps

**Figure 1:**
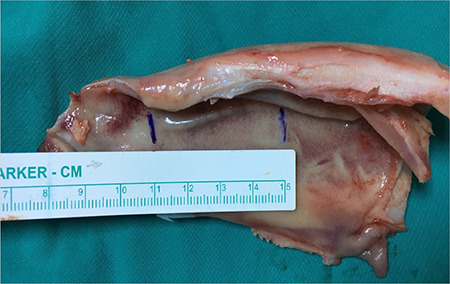
Sheep nasal septum. The region that was prepared for the experiment is marked with vertical blue lines

**Figure 2:**
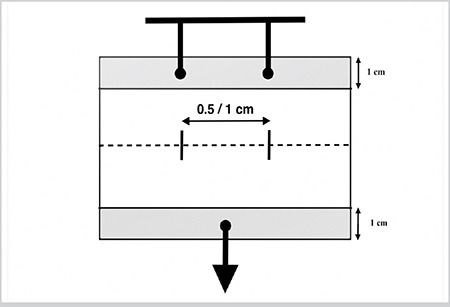
Schematic representation of the HS_max_ measurements. Horizontal dashed line symbolizes the laceration. Vertical thin solid lines crossing the dashed line symbolize the sutures. Arrow stands for the Newton-meter and shows the direction of the tensile force applied HS_max_: Maximum tissue holding strength, cm: Centimeter

The nasal septum was completely removed from the sheep head. The thickness of the mucosal-perichondrial layer varies throughout the craniocaudal axis of the sheep’s nasal septum. The most caudal part of the nasal septum is covered with the thickest layer of mucosa-perichondrium and the thickness of the mucosal-perichondrial wall decreases as it advances cranially. To enable standardized measurements, the middle third section of the septum, i.e., the region between the 4^th^ and 8^th^ cm from the most caudal part of the septum, was used (Figure 1). Bilateral mucosal-perichondrial flaps were elevated from the cartilage septum, except for the most dorsal and ventral 1 cm-height parts. The flaps were not raised along the dorsal and ventral borders of the septum, keeping the 1 cm-height mucosal-perichondrial attachments to the underlying cartilage intact. Septal cartilage between those superior and inferior 1 cm-height edges was removed. The upper cartilage strip that was left intact was then longitudinally cut into two equal parts, resulting in two mucosal-perichondrial leaves on either side of the septal base. A horizontal incision was made through the mid-height of the mucosal-perichondrial leaf to simulate a tear. The incision was then sutured with two interrupted sutures using one of the four suture materials described below and leaving either 5 mm or 10 mm distance between the sutures (Figure 2). Non-identical four-throw sliding knots were applied for each suture since these are widely used in septum surgery. A 3 mm suture tail was left on each side of the knot. The same surgeon tied all knots for each suture type, size, and distance.

### Suture Groups

To assess the effect of the type and diameter of different suture materials to the HS_max_, 4-0 Polyglactin 910 (Vicryl Rapide, Ethicon, Johnson & Johnson Medical N.V., Belgium), 5-0 Polyglactin 910 (Vicryl Rapide, Ethicon, Johnson & Johnson Medical N.V., Belgium), 4-0 Polydioxanone (PDS II, Ethicon, Johnson & Johnson Medical N.V., Belgium) and 5-0 Polydioxanone (PDS II, Ethicon, Johnson & Johnson Medical N.V., Belgium) were used. A 16-mm curved cutting needle was used in all suture materials. To determine the effect of the distance between two adjacent sutures on HS_max_, spaces of 5 mm and 10 mm were left between the adjacent sutures for each suture material. Consequently, HS_max_ measurements were taken from eight groups, i.e., 4-0 PDSII (5mm), 4-0 PDSII (10 mm), 5-0 PDSII (5 mm), 5-0 PDSII (10 mm), 4-0 Vicryl Rapide (5 mm), 4-0 Vicryl Rapide (10 mm), 5-0 Vicryl Rapide (5 mm), and 5-0 Vicryl Rapide (10 mm). At least 16 measurements were made for each suture group of mucosal-perichondrial flaps. We excluded mucosal-perichondrial flaps with undesired tears, excessive elevation, and broken cartilage strip during the preparation.

### HS_max_ Measurements

**Figure 3:**
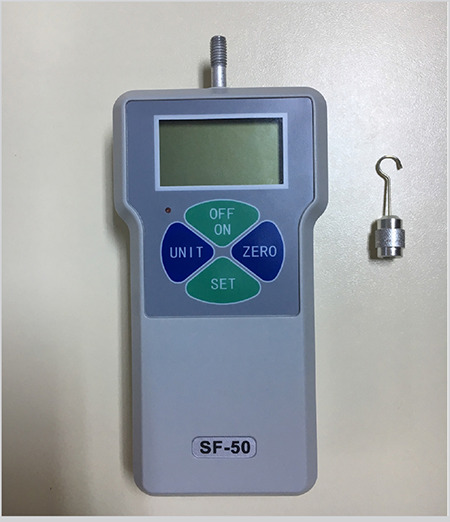
Digital Newton-meter that was used for measurements

The HS_max_ were measured and quantified in Newton units using a commercially available portable digital Newton-meter (SF50, Geratech, China) (Figure 3). Two towel forceps were fixed into one of the cartilage strips. The hook of the Newton-meter was placed into the middle of the other cartilage strip. Holding the tissue sample with the aid of a Cottle elevator and pulling the Newton-meter downwards, tensile force was applied to the sutures (Figure 2). This measurement method is similar to that used in a recent study on tissue holding strength of porcine kidney (7). All measurement procedures were recorded on video using the digital camera of a cellphone (iPhone^®^7 Plus, Apple Inc, Cupertino, California, USA) by one of the authors (A.K). The video records were then analyzed in the computer environment (iMovie, Apple Inc.) by another author (O.E) who did not participate in the video recording process. The values that had been read on the screen of the Newton-meter at the time points when the force caused opening of the knot or tearing of the mucosal-perichondrial leaflet were noted.

### Statistical Analysis

Statistical analyses were performed using IBM SPSS for Windows software (IBM Corp. Released 2013. IBM SPSS Statistics for Windows, Version 22.0. Armonk, NY, USA). The number of nasal septum specimens was determined by performing power analysis and it was found that at least 16 sheep nasal septal flaps should be included for each suture group in the study when 90% significance and α=0.05 level was calculated. Continuous variables were presented as mean ± standard deviation values. The Independent Samples t-test was used to compare the means of two independent groups. Comparisons of the mean holding strength of the same suture material at different distances were analyzed with Mann-Whitney U test. A value of p<0.05 was considered statistically significant. Linear regression analyses were performed to determine the strength of predictors, and p values <0.05 were considered statistically significant.

## Results

The mean HS_max_ values for each suture group are given in Table 1. The highest (12.74±2.40 N), and lowest (6.74±3.03 N) mean HS_max_ values were detected in the 5-0 Vicryl Rapide (5 mm), and 4-0 PDS II (5 mm) groups, respectively.

The Independent Samples t-test was applied to assess the effect of suture material, suture diameter and suture distance on HS_max_. The mean HS_max_ value for polyglactin 910 and polydioxanone sutures was 11.44±3.41 N and 8.26±3.34 N, respectively (p<0.001). The mean HS_max_ value for 5 mm and 10 mm suture distance was 8.98±3.81, and 10.71±3.45, respectively (p=0.04). Linear regression analysis also showed that the HS_max_ was greater for polyglactin 910 sutures and at 10 mm suture distance (Table 2). There was no statistically significant difference between the mean HS_max_ values of sutures with 4-0 (9.22±3.67 N) and 5-0 (10.47±3.69 N) diameters (p=0.057). Additionally, comparisons of the mean holding strengths of the same suture material for 5 mm and 10 mm suture distance are given in Table 3.

Tensile force resulted in the opening of the knots or tearing of the mucosal-perichondrial flaps. PDS II and Vicryl Rapide sutures were compared in terms of sliding of the knots. Tensile force resulted in the opening of the knots in 35.9% (n=23) of the polydioxanone and 6.2% (n=4) of the polyglactin 910 sutures, respectively (p<0.05).

## Discussion

Different tissues show varying resistance to stress. Several studies have measured the maximum resistance strength before tears in some tissues and organs (6, 7, 8). Rodrigues et al. (8) measured HS_max_ values in 10 pigs using 3-0 vicryl in eight intra-abdominal organs (fascia, aorta, vena cava, peritoneum, small and large bowel, uterus, and fallopian tube). The most resistant tissue was the fascia at 11.43 N, and the weakest was the fallopian tubes at 1.25 N. Thus, it was determined what force surgeons could apply to which tissues during suturation in laparoscopic surgery. Tarin et al (9) made HS_max_ measurements of various sutures in the renal capsule. Hem-o-lok clips were found to be more resistant and their use was recommended. In our study, HS_max_ measurements were made in the sheep nasal septum for two different suture materials, two different suture diameters and two different suture spacings. It was determined that the use of polyglactin 910 as the suture material and 10 mm as the suture spacing resulted in higher HS_max_ values. In other words, polyglactin 910 should be preferred as the suture material and 10 mm space should be left between adjacent sutures when repairing nasal septal mucosal lacerations. Although the mean HS_max_ value of 5-0 sutures (10.47 N) was higher than that of the 4-0 sutures (9.22 N), there was no statistically significant difference between the two sutures in terms of suture diameter (p=0.057).

The PDS II monofilament suture group was observed to have a greater tendency to slippage and opening, compared to the Vicryl Rapide suture group. This showed that even if PDS II sutures used in septal mucosal-perichondrial laceration repair remain for a long time without hydrolysis, they are less reliable in the short-term. PDS II sutures are more slippery as they are monofilament, and because of their structural properties, if monofilament sutures are to be used, it can be considered more appropriate to apply more than 4 knots. Trimbos et al. (10) evaluated the performance of opening knots in multifilament and monofilament suture materials. Non-identical and parallel sliding knots were reported to differ little in respect of knot reliability. In addition, five-throw knots were significantly stronger than three-throw knots if monofilament suture material was used (11, 12). In our study, non-identical sliding knots with four throws were used to prevent the knots opening.

Apart from Vicryl Rapide and PDS II, there are many types of absorbable monofilament and multifilament sutures that can be used inside the nose, such as Caprosyn (Polygytone 6211), Monocryl (Poliglecaprone 25), Maxon (Polyglyconate), and PGA (Polyglycolic acid). Each of these suture types has advantages and disadvantages in respect of the inflammatory reaction created in the tissue, the time to absorption, tensile strength, and suture reliability. Retrieving sutures used in septal mucosal-perichondrial laceration repair from the nose is an extremely challenging procedure and so absorbable sutures are preferred in the majority of cases, as they do not require long-term care. A good suture must be biocompatible, be able to be effectively absorbed, resistant and appropriate to the organ or area where it is to be used (13). Two types of suture that are often used in septal mucosal-perichondrial laceration and septal perforation repairs are Vicryl Rapide (polyglactin 910) and PDS II (Polydioxanone). In our study, Vicryl Rapide and PDS II suture types were evaluated at the two different diameters of 4-0 and 5-0. As the absorption time of PDS II is longer than that of Vicryl Rapide and the study results showed a lower HS_max_ value for PDS II, it can be said that the use of Vicryl Rapide is more advantageous in the repair of septal mucosal-perichondrial lacerations. In our clinical practice, we prefer 4-0 Vicryl Rapide or 5-0 Vicryl Rapide suture types and we place the stitches at 10 mm distance in the repair of nasal septal mucosal-perichondrial tears.

In the literature, there is no objective information about which material should be applied at which distance in the repair of nasal septal mucosal-perichondrial lacerations. In our study, two different suture distance groups were formed as 5 mm and 10 mm to examine whether the suture spacing had any effect on the HS_max_ alue. The HS_max_ values measured at 10 mm spacing were seen to be higher than those measured at 5 mm spacing. This might be an inherent result of using closer sutures, which in turn caused more tissue violation. Using the proper suture material, size, and the proper distance between two adjacent sutures, will potentially, to a great extent, reduce septal perforation rates and increase quality of life for patients. Furthermore, extensive studies would be useful to examine the HS_max_ value of different types of sutures in the repair of tears in nasal septal mucosal-perichondrial flaps.

There were some limitations to this study. In a postmortem study by Rosen et al. (14), tissue resistance was shown to be significantly weaker than the results of *in vivo* studies.Although the use of postmortem tissue could be seen as a limitation of the current study, the aim of the study was not to measure the exact tissue resistance of the sheep septum, but to compare the effect of different types of sutures on holding strength. Therefore, the use of postmortem tissue can be considered not to have affected the results. Sheep septum was selected as a model for the practical reasons of ease of availability and because sheep nasal septal flaps are comparable to human nasal septal flaps in terms of resistance, ease of mucosal-perichondrial elevation, size, and thickness. Additionally, tissue resistance of this study was measured at room temperature and not measured at “living” tissue temperature, and physiological events such as airflow, crusting, inflammatory reactions will not occur in postmortem tissue. Since the results of this study represent the outcomes obtained from a postmortem animal experiment at best and should be confirmed by further *in vivo* and/ or fresh human cadaver studies.

## Conclusion

The type of suture material and the distance between sutures should be taken into consideration when repairing nasal septal mucosal-perichondrial lacerations. Polyglactin 910 suture material with 10 mm spacing between two adjacent sutures may be more durable than the other suture materials when repairing nasal septal mucosal lacerations.

**Main Points**

• Appropriate repair of nasal mucosal lacerations is essential to prevent the development of septal perforation during the healing process.

• The PDS II (polydioxanone) monofilament suture group was observed to have a greater tendency to slippage and opening, compared to the Vicryl Rapide (polyglactin 910) suture group.

• Using Vicryl Rapide (polyglactin 910) with a distance of 10 mm in repair of mucosal-perichondrial flaps may be more appropriate than other suture materials and suture distances.

## Figures and Tables

**Table 1:**
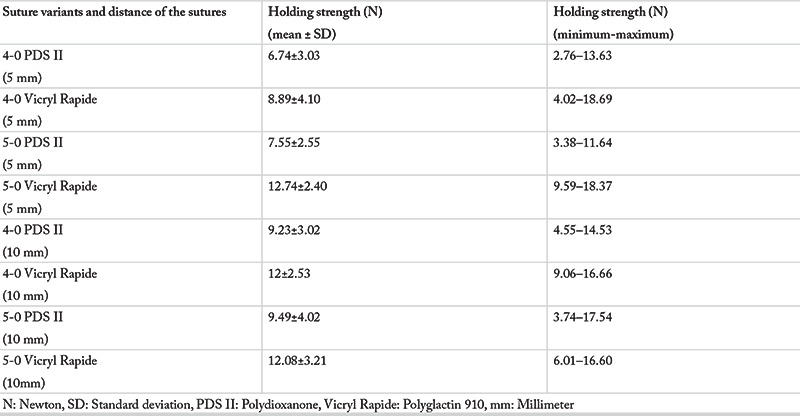
Mean holding strength according to suture variants and suture distance

**Table 2:**
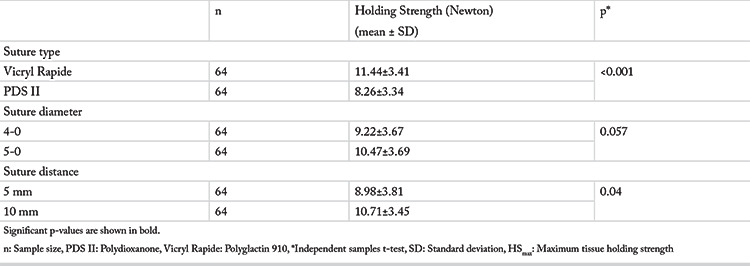
Comparison of suture types, diameters, and distances in terms of mean HS_max_

**Table 3:**
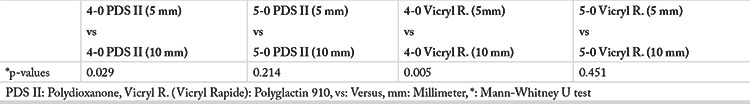
Comparisons of the mean holding strength of the same suture material at different distances
